# Systematic analysis of factors that improve homologous direct repair (HDR) efficiency in CRISPR/Cas9 technique

**DOI:** 10.1371/journal.pone.0247603

**Published:** 2021-03-05

**Authors:** Mariateresa Di Stazio, Nicola Foschi, Emmanouil Athanasakis, Paolo Gasparini, Adamo Pio d’Adamo

**Affiliations:** 1 Institute for Maternal and Child Health-IRCCS “Burlo Garofolo”, Trieste, Italy; 2 Department of Medicine, Surgery and Health Sciences, University of Trieste, Trieste, Italy; CNR, ITALY

## Abstract

The CRISPR/Cas9 bacterial system has proven to be an powerful tool for genetic manipulation in several organisms, but the efficiency of sequence replacement by homologous direct repair (HDR) is substantially lower than random indel creation. Many studies focused on improving HDR efficiency using double sgRNA, cell synchronization cycle, and the delivery of single-stranded oligo DNA nucleotides (ssODN) with a rational design. In this study, we evaluate these three methods’ synergistic effects to improve HDR efficiency. For our tests, we have chosen the *TNFα* gene (NM_000594) for its crucial role in various biological processes and diseases. For the first time, our results showed how the use of two sgRNA with asymmetric donor design and triple transfection events dramatically increase the HDR efficiency from an undetectable HDR event to 39% of HDR efficiency and provide a new strategy to facilitate CRISPR/Cas9-mediated human genome editing. Besides, we demonstrated that the *TNFα* locus could be edited with CRISPR/Cas9 methodology, an opportunity to safely correct, in the future, the specific mutations of each patient.

## Introduction

Over the last decade, the novel CRISPR-associated endonuclease Cas9 protein has been used for therapeutic and analytical approaches in a vast spectrum of cell types and animal model [1, 2]. Thanks this method, the genes editing has become as simple, fast, and economical as never before.

The CRISPR/Cas9 methodology exploits the ability of the bacterial protein Cas9 to induce DNA double-strand breaks (DSB) in a trinucleotide region repeated in the genome, motif PAM region, directed by a 20–22 bp synthetic RNA sequence (gRNA) located next to PAM [1]. DSB activates the cell’s repair mechanisms, including non-homologous end joining (NHEJ) and homologous direct recombination repair (HDR) to repair the DNA strands. NHEJ produces insertions and deletions in the DNA target sequence, creating a frameshift that suppress the correct protein production, generating a knock-out model; while HDR has been generally used to editing DNA coding or non-coding regions by introducing specific DNA sequences (donor DNA) in the proper locus [3–9].

In the fast-evolving field of gene editing using CRISPR/Cas9, crucial efforts to improve the efficiency of HDR have been made; but the reported success rates remained very low in many cell type and in vivo studies [5, 10, 11].

So far, many published protocols have explained how to improve the efficiency of HDR [1, 5, 12]. Acosta et al. described a highly efficient HDR targeting approach based on the use of two sgRNAs (single guide RNA) flanking the targeted region in mouse ESC lines by a new method called “two gRNA-driven homozygous HR” [1]. Zhou et al. reported that using two sgRNAs increases the endogenous gene, targeting mouse cells’ efficiency [1, 5].

Other authors tried to improve the HDR by blocking the cell cycle at different phases, showing that treatment with nocodazole increases HDR’s efficiency. Nocodazole blocks cells at the G2/M phase when DNA is totally replicated, and the nuclear membrane is broken, allowing Cas9 to access DNA and enhancing the HDR [5]. Richardson et al. focused on understanding how the Cas9 enzyme interacts, cuts, and separates from the DNA target in physiological conditions to improve the genome editing by HDR event [12]. They showed that the enzyme locally releases the PAM-distal non-target strand after cleavage but before complete dissociation, making this strand available for complementary annealing of single-stranded oligo DNA nucleotides (ssODN). For this reason, the ssODN with asymmetric arms with the short arm of 36bp on the PAM-distal side and the long arm of 91bp on the PAM-proximal site of the break, complementary to non-target DNA strand, show the highest efficiency of HDR [12].

These results led us to investigate the effect on HDR, combining the three approaches described. We wondered if the three protocols synergically increase the efficiency of HDR.

For this aim, we have chosen the *TNFα* gene (NM_000594) for its crucial role in various biological processes and diseases. *TNFα* encodes a multifunctional proinflammatory cytokine that belongs to the tumor necrosis factor superfamily. This cytokine is involved in regulating a broad spectrum of biological events, such as immune cell regulation, proliferation, differentiation, apoptosis, lipid metabolism, coagulation and thus is implicated in a variety of diseases in adults and children [13–15]. *TNFα* is also involved in rheumatoid arthritis (RA), a complex autoimmune disease, with a relatively constant prevalence of 0,5–1% in the world’s populations that affect many organs, including kidney, eyes, spleen, heart, and lungs [16]. Besides, *TNF*α can alter insulin response regulation, and its expression affects the pathophysiology of pediatric insulin resistance [17, 18]. Recent studies focused their attention on the role of the proinflammatory cytokine tumor necrosis factor in heart failure development, showing a direct relationship between the level of *TNFα* expression and the severity of heart disease [19]. Furthermore, this cytokine has relevance in tumor immune surveillance and is crucial in tumor development and progression [13].

Based on this scenario, the possibility of modifying this gene became a fascinating and intriguing objective to safely correct, in the future, the specific genetic variants of each patient. Here we report a robust and straightforward approach to improve the efficiency in editing this gene, and it is also a proof of concept for a method that can be used more extensively for other genetic sequences.

## Material and methods

### Design of the sgRNAs and plasmid constructions

According to the following criteria, two different sgRNAs were designed: close location to the mutation(s), following PAM at the 3’end, and 20-nt length. The sgRNA were purchased from IDT (Integrated DNA Technologies, IDT, USA). The crRNAs were already cloned in pX333-U6-Chimeric_BB-CBh-hSpCas9 (Addgene, plasmid ID #42230). The two opposite BbsI and BsaI restriction sites were used to insert the guides under a U6 promoter’s control. For this purpose, self-complementary oligonucleotides (Integrated DNA Technologies, IDT, USA) were annealed by gradual cooling with prior denaturalization at 94° C. The duplex oligonucleotides also presented cohesive ends with the 3’ overhangs left after pX333 incubation with the BbsI and BsaI restriction enzymes, serving for the ligation of the insert-plasmid with T4 DNA ligase (EL0014; Thermo Fisher Scientific, Waltham, MA, USA). Moreover, the sgRNA1 and sgRNA2 were cloned in the plasmid pX459 (Addgene, plasmid ID #62988), which carried the puromycin antibiotic resistance. DH5α competent cells were transformed with each of the plasmid constructs for ampicillin selection and amplification in liquid culture. The vectors were purified using a HiSpeed Plasmid Midi Kit (QIAGEN, Hilden, Germany) and were Sanger sequenced to verify the specific crRNA inserts’ correct cloning.

### HEK293 cell culture and transfections

HEK293 cells were maintained in DMEM (Invitrogen) supplemented with 10% heat-inactivated fetal bovine serum (FBS), 100 U/mL penicillin, and 100 μg/mL streptomycin at 37°C under 5% CO_2_. The 5x10^5^ cells were transfected with 3 ug of plasmids. Fugene transfection reagent (Promega). For the directed editing, 3 ug of plasmid was delivered to 5x10^5^ HEK293 cells with 1 uL of the pertinent ssODN at 10 μM (10 pmol). The nocodazole was added after transfection at a concentration of 100 ng/mL for 24 h. Each Cas9-gRNA vector was co-transfected in HEK293 cells with an empty CRISPR vector coding for a GFP green fluorescent protein (pX458 Addgene, plasmid ID #48138) in order to monitor the transfection efficiency. All experiments all experiments were performed 7 to 9 times to ensure robustness of the results.

### PCR amplification of the target region

A 1286 nt region of *TNFα* locus containing the target site, were PCR amplified using the following primer sets. The target locus was amplified for 35 cycles with specific forward (TNF-X-Fw:5’-CGCCACCACGCTCTTCTG-3’) and reverse (TNF-Alw-Rv:5’-CGGTTCAGCCACTGGAGC-3’) primers targeting exon 1 and exon 4 of the *TNF*α gene, respectively. The PCR reaction was performed using 50 ng of genomic DNA and Kapa Hot start high-fidelity polymerase (Kapa Biosystems, Wilmington, MA) in a high GC buffer according to the manufacturer’s protocol. The thermocycler setting consisted of one cycle of 95°C for 5 min, 30 cycles of 98°C for 20 s, 61°C for 15 s and 72°C for 30 s, and one cycle of 72°C for 1 min. The PCR products were analyzed on 1,5% agarose gel containing Midori Green Xtra (NIPPON Genetics Europe, Dueren, Germany). About 200 ng of PCR DNA was used for T7 endonuclease I and SmaI digestion analyses.

### T7-endonuclease I assay

The widely used T7-endonuclease I assay targeted and digested hetero-duplexes formed by the hybridization of mutant and wild-type (wt) strands resulting in two smaller fragments; this method was performed to assess sgRNA-specific activity. After the transfection, the HEK293 cells were incubated for 48 hr. The cells were then pelleted, and the lysis performed using the QIAamp DNA Mini Kit (Qiagen). The PCR products were denatured and then reannealed using the following program: 95° C for 5 min, ramp down to 85°C at 2°C/s and ramp down to 25°C at 0.1 C/s. After the reannealing step and the consequent heteroduplex formation, 5 units of T7 endonuclease I (New England Biolabs, Ipswich, MA) were added to the mix and incubated 1 hr at 37°C. The product was resolved on 1,5% agarose gel containing Midori Green Xtra (NIPPON Genetics Europe, Dueren, Germany).

### Clonal amplicon sanger sequencing

A PCR product obtained from the target locus was cloned utilizing a kit for sequencing purposes (TOPO TA Cloning Kit, Thermo Fisher Scientific) and introduced in *E*. *coli*. Sanger sequencing was used to sequence 10 individual colonies to reveal the clonal genotype and then the general indels and HDR frequency.

### Design of the ssODNs

Single-stranded donor oligonucleotide (ssODN) for the HDR was designed with symmetric (60nt long) and asymmetric (90nt, 36nt long) homology arms in both orientations (5’-3’;3’-5’), complementary to target and non-target DNA strand. The ssODNs sequence included a new and unique SmaI restriction site, juxtapose to the homology arms. These ssODNs were synthesized as Ultramer Oligonucleotides (Integrated DNA Technologies, IDT). Since the edited sequence contained a newly acquired SmaI restriction site, PCR products were restricted immediately after SmaI enzyme digestion (NEB, Ipswich, MA).

### Analysis of HDR by SmaI restriction digestion

The reaction consisted of 1.5 μg of PCR DNA and 5 units of SmaI enzyme in CutSmart Buffer (NEB, Ipswich, MA). After 1hr of incubation at 37°C, the reaction was arrested with heat inactivation at 65°C for 20 min. The product was resolved on 1.5% agarose. The band intensity was quantitated through the Image Lab program. The percentage of HDR was calculated using the following equation (b / a + b) × 100 for the single cut strategy, (c + d / a + b + c + d) x 100 for the dual cut strategy. In the first equation, ‘a’ is the band intensity of DNA substrate and ‘b’ and ‘c’ are the cleavage products. In the second equation, ‘a’ is the band intensity of DNA substrate wild type, ‘c’ and ‘d’ are the cleavage products, and ‘b’ is the deleted fragment in which both sgRNA worked properly but there was not an HDR event. To further confirm the edited sequence’s presence, conventional Sanger sequencing was performed (S3A Fig).

### Off-target analysis

To predict the most likely off-target sites for the sgRNAs used to knock-down the *TNF*α gene in this study, we adopted a public webserver: (https://eu.idtdna.com/site/order/designtool/index/CRISPR_PREDESIGN) able to assess and prioritize potential CRISPR/Cas9 activity at off-target loci based on predicted positional bias of a given mismatch in the sgRNA protospacer sequence and the total number of mismatches to the intended target site. The CRISPR design tool (IDT) scored a total of 202 (101 for sgRNA1 and 101 for sgRNA2) potential off-target sites in the human genome. The off-targets were evaluated between 0 to 100, where the higher number indicated a lower chance of off-target presence. The top three potential off-target sites (11 ≤ scores ≤ 26) for each sgRNA, and the first genomic locus, regardless of its position in the off-target list in the database, were assessed by T7 endonuclease assay in HEK293 cells.

## Results

### Genome editing of the human *TNFα* gene

Intending to improve the efficiency of HDR, we tested and further combined three strategies already reported in the literature able to increase the HDR efficiency but never used together [1, 5, 12]. We wondered if these protocols (use of two sgRNA, rational design of ssODNs, and cell synchronization) could work in synergy to improve the HDR efficiency. In parallel, we added a fourth condition in which the cells were transfected three consecutive times.

To this end, we proceeded in two steps:

1. First, to increase the efficiency of excision of the Cas9 nuclease, we explored and compared the use of one sgRNA (which induced one DSB) and dual sgRNAs (who determined two DSB located in the region of the *TNFα* gene.
2. Next, after choosing the best sgRNA strategy (with one or two sgRNA), we compared the use of various donor ssODNs, with different structure, in synchronized and unsynchronized cells with single and multiple transfection events.

### Selection of specific RNA guides for DSB induction

**Fig 1:**
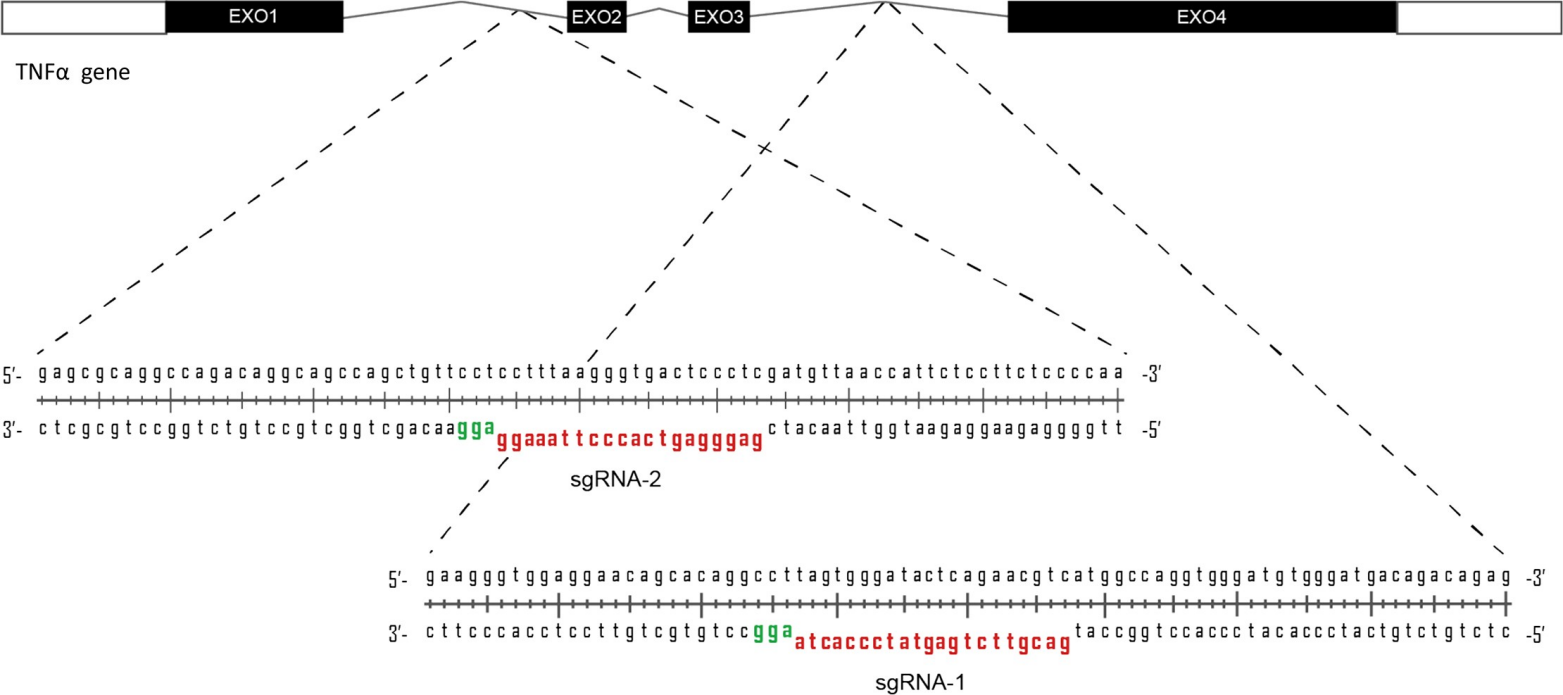
CRISPR/Cas9 targeting of the human *TNF*α gene. Schematic representation of human *TNF*α gene. The magnified view illustrates the sgRNAs (in red) and the PAM sequences (in green).

For this purpose, we used the previously described *Staphylococcus pyogenes* nuclease, which utilized a human-codon optimized SpCas9 and a chimeric sgRNA expression vector to direct efficient site-specific gene editing [6, 20]. We designed two 20-nt long sgRNA with a wide-spam of 473 nucleotides to guide Cas9 to introns 1 and 3 of the *TNF*α gene (sgRNA1 and sgRNA2) (Fig 1). Without donor DNA containing the homology arms, cells repair the DNA primarily by NHEJ, creating insertion and deletion (indels) [20]. Considering the size of the small introns (≈300-600bp) in the *TNF*α locus, the indels could involve adjacent exons and could generate frameshift mutations that knocked-out the *TNFα* gene.

In detail, in pX333 plasmid, the two sgRNAs were cloned as a single guide (pX333-sgRNA1; pX333-sgRNA2), in tandem combination (pX333-2sgRNA1/1; pX333-2sgRNA2/2), and the two different guides were cloned in the same vector (pX333-2sgRNA1/2). To assess whether the puromycin plasmid could help positive clone selection, we cloned only the two sgRNAs in the pX459 vector (pX459-sgRNA1 and pX459-sgRNA2), a plasmid that carried the puromycin resistance. The HEK293 cells were transfected with the seven constructs thus obtained, and the puromycin antibiotic was added only in cells transfected with the pX459 vector.

**Fig 2:**
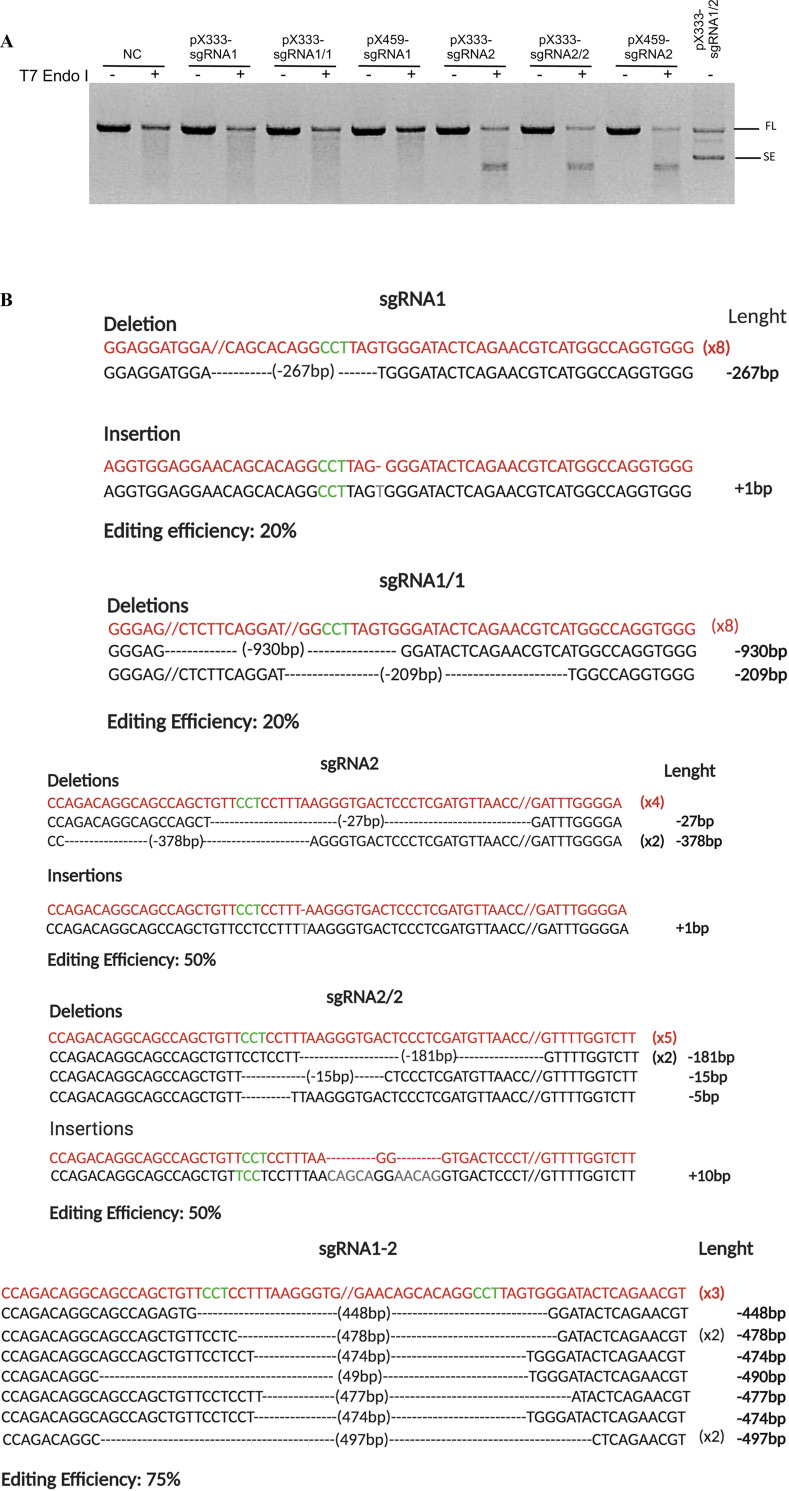
NHEJ-mediated knock-out of human *TNFα* gene using the CRISPR/Cas9 system. **(A)** The T7EI nuclease assay on *TNFα* gene showed targeted cleavage of the digested PCR products in HEK293 cells transfected with pX333-sgRNA1, pX333-sgRNA1/1, pX459-sgRNA1, pX333-sgRNA2, pX333-sgRNA2/2, pX459-sgRNA2 and pX333-sgRNA1/2. Cells transfected with 2sgRNA shows the short edited PCR product. (not determined, ND; negative control, NC; full-length, FL; short-edited, SE). **(B)** Sequence analysis of PCR products surrounding the Cas9 target sites in the genome of HEK293 transfected with the same plasmid (in bold) showed a wide variety of Indels mutations mediated by NHEJ. The top sequence in red is the unmodified sequence; in green are the PAMs. The mismatches/insertions are indicated in gray. The number of PCR amplicons for each sequence is indicated in parentheses, and the modified length is indicated.

To evaluate the efficiency of DNA cutting and NHJE repair after using sgRNA guides, the genomic DNA was isolated from HEK293 cells and screened for the presence of site-specific gene modification by PCR amplification and T7E1 endonuclease assay of the region around the target sites (Fig 2A). The results showed detectable digestion bands in pX333-sgRNA2, pX333-sgRNA2/2, pX459-sgRNA2. The method of sgRNAs cloned in single or in tandem when co-expressed with the SpCas9 nuclease could mediate gene modification with a comparable efficiency level. No differences were finally detected between pX333 or pX459 plasmids after puromycin selection, possibly due to the high transfection efficiency obtained in HEK293 cells. Notably, HEK293 cells transfected with the 2sgRNA plasmid (2sgRNA1/2) and not treated with T7E1 nuclease resulted in full-length (FL) and in short-edited (SE) amplicons confirming the expected deletion of the region between the two selected protospacers (Fig 2A).

Moreover, the frequency of indels in the cells transfected with all sgRNAs was measured by sequencing 10 PCR amplicons encompassing the target sites. As reported in Fig 2B, the highest editing frequencies achieved were 50% and 75% using sgRNA2 and 2sgRNA1/2 guides, respectively (Fig 2B). At this locus, the insertions and deletions presented variable patterns of rearrangements of the coding sequence, insertion from 1 to 10 nucleotides, and deletion from 5 to 930 nucleotides. The region’s deletion between the 2 PAMs was observed in the cells transfected with 2sgRNA plasmid (2sgRNA1/2) (~480nts).

Furthermore, we recognized the predominance of a precise junction between the two DSBs when 2sgRNA1/2 was transfected, a mechanism already described [1, 21, 22].

These data confirmed that the dual sgRNA (2sgRNA) was the most efficient DNA excision method in the endogenous locus. We selected the sgRNA2 and the 2sgRNA1/2 guides for the following HDR editing based on these genomic results.

### HDR efficiency

We chose the plasmids pX333-sgRNA2 and the pX333-2sgRNA1/2 that showed the highest degree of DSB in HEK293 transfection for HDR. Traditional HDR gene editing required long homology arms to allow proper and high-specificity recombination. The use of Cas9-gRNAs directed recombination allows the use of much smaller homology arms (~90 bp to 700 bp) with higher recombination rates than conventional HDR [1].

We decided to assess HDR efficiency by transfection of single (pX333-sgRNA2) and double sgRNA (pX333-2sgRNA1/2) coupled with a rational ssODNs design. The structure of ssODNs with asymmetric arms complemented to a non-target locus with a long arm on the PAM-proximal side, and a short arm on the break’s PAM-distal side has been previously reported to induce the highest HDR efficiency [12]. However, we decided to test this asymmetric donor for the *TNFα* locus by comparing it with other possible ssODN structures on HDR efficiency.

**Fig 3:**
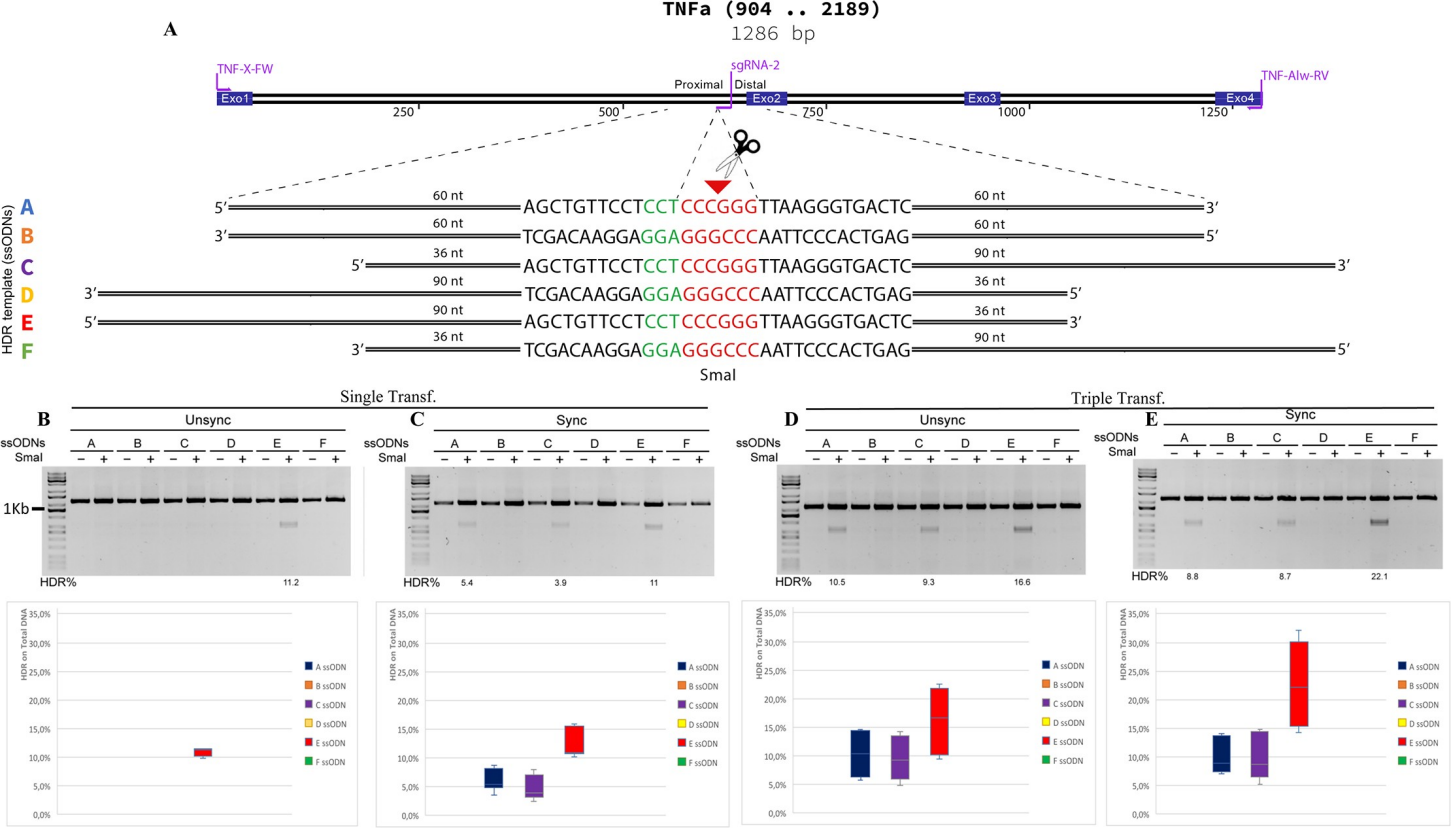
Systematic investigation of DNA templates for efficient HDR at the *TNFα* locus in HEK293 cells. **(A)** A segment of human *TNFα* shows the genome structure, the sgRNA2 guide site, and the primer used for PCR amplification (in violet). +1: ATG. Six HDR templates (color-coded) were tested for HDR efficiency, the PAM region (in green). Template ssODN contains SmaI restriction sites (in red) flanked by various lengths of homology arms. **(B)** HDR efficiency was tested in single and triple transfection, in combination with synchronized cells. The mean % HDR and standard deviation (error bar) were determined by bands quantification after SmaI digestion from seven and eight replicates. Representative gels from PCR and HDR analyses are shown for each cell condition. Not determined, ND.

Therefore, we generated twelve ssODN molecules with different sequences overlapping on the 5’ and 3’ side of the break, specific for pX333-sgRNA2 and pX333-2sgRNA1/2 guides and complementary to the target or non-target DNA strand (Figs 3A and 5A). We co-delivered these ssODNs combined with a single sgRNA and for the first time with two sgRNAs (2sgRNA1/2).

To facilitate the selection of HDR events, we inserted a restriction site for the enzyme SmaI in all the ssODNs. The pX333-sgRNA2 and pX333-2sgRNA1/2 plasmids and the respectively six ssODNs were then co-transfected in HEK293 cells. Besides, we introduced a nocodazole cells synchronization, described to improve the HDR [5]. Then, we included a fourth condition in which the cells were transfected three consecutive times with the same donor and plasmid.

Since the edited sequences contained a newly acquired SmaI restriction site, the HDR efficiency could be easily detected using the SmaI digestion on PCR products obtained with primers flanking the targeted locus.

### Single cut and HDR efficiency

First, we determined the HDR efficiency with the co-delivery of pX333-sgRNA2 guide (that induces single DSB) and the six ssODN molecules (Fig 3A to 3E). Since the ssODN had the SmaI digestion site, we compared the HDR efficiency after SmaI digestion in single transfection events, triple transfection, with and without nocodazole cells treatment. With SmaI digestion, after PCR amplification, we could identify the HDR events’ efficiency by bands DNA quantification. Notably, among the six ssODNs employed, the donor E is the only one that showed SmaI digestion and so HDR event in single transection events in unsynchronized cells (Fig 3B). Donors A and C were able to induce HDR but only after nocodazole treatment with a low frequency of 5,4% and 3,9%, respectively. With triple transfections in unsynchronized cells, donor A increased the HDR efficiency to 10,5%, and donor C to 9,3%. The highest HDR frequency achieved was 22.1% for donor E, with triple transfection and nocodazole cells treatment (Fig 3B to 3E). The tree donors, A, C, and E, are all complementary to the non-target DNA strand, and this observation is consistent with previous studies [12].

The graphic in S1A and S1B Fig compared the HDR efficiency in synchronized and unsynchronized cells in one and triple cell transfections. The graphic in S1C and S1D Fig instead, compared the HDR efficiency between single and triple transfection in synchronized and unsynchronized cells. These data showed that no significant differences have been detectable with nocodazole treatment in our experimental condition, while the triple transfection increases the HDR efficiency, but only for the ssODNs A, C, and E. While the other three donors (ssODN B, D, F), characterized to be complementary to the target DNA strand, did not induce HDR events.

**Fig 4:**
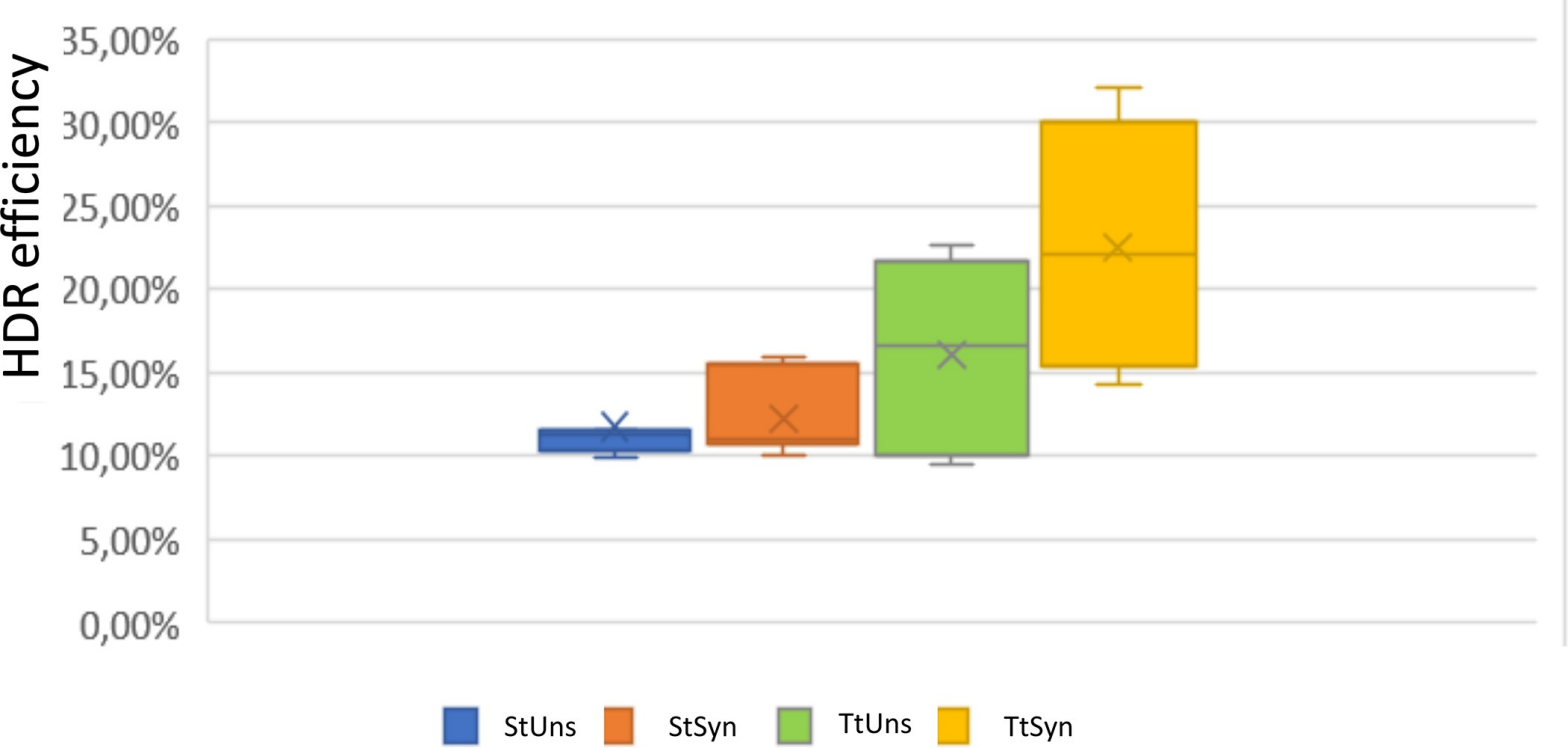
Graphic comparison of HDR efficiency of donor E. The triple transfection and nocodazole treatment increased the HDR efficiency by about two-fold. StUns, single transfection in unsynchronized cells; StSyn, single transfection in synchronized cells; TtUns, triple transfection in unsynchronized cells; TtSyn, triple transfection in synchronized cells.

Notably, donor E induced the highest HDR efficiency. It showed the same structure described by Richardson et al. to be the best for the HDR, with asymmetric arms complementary to a non-target locus with 90bp on the PAM-proximal side and 36bp extension arm on the PAM-distal side of the break [12]. The HDR efficiency using donor E increased up to 22% (two-fold) with triple transfection in synchronized cells (Fig 4).

### Dual cut and DSB efficiency

**Fig 5:**
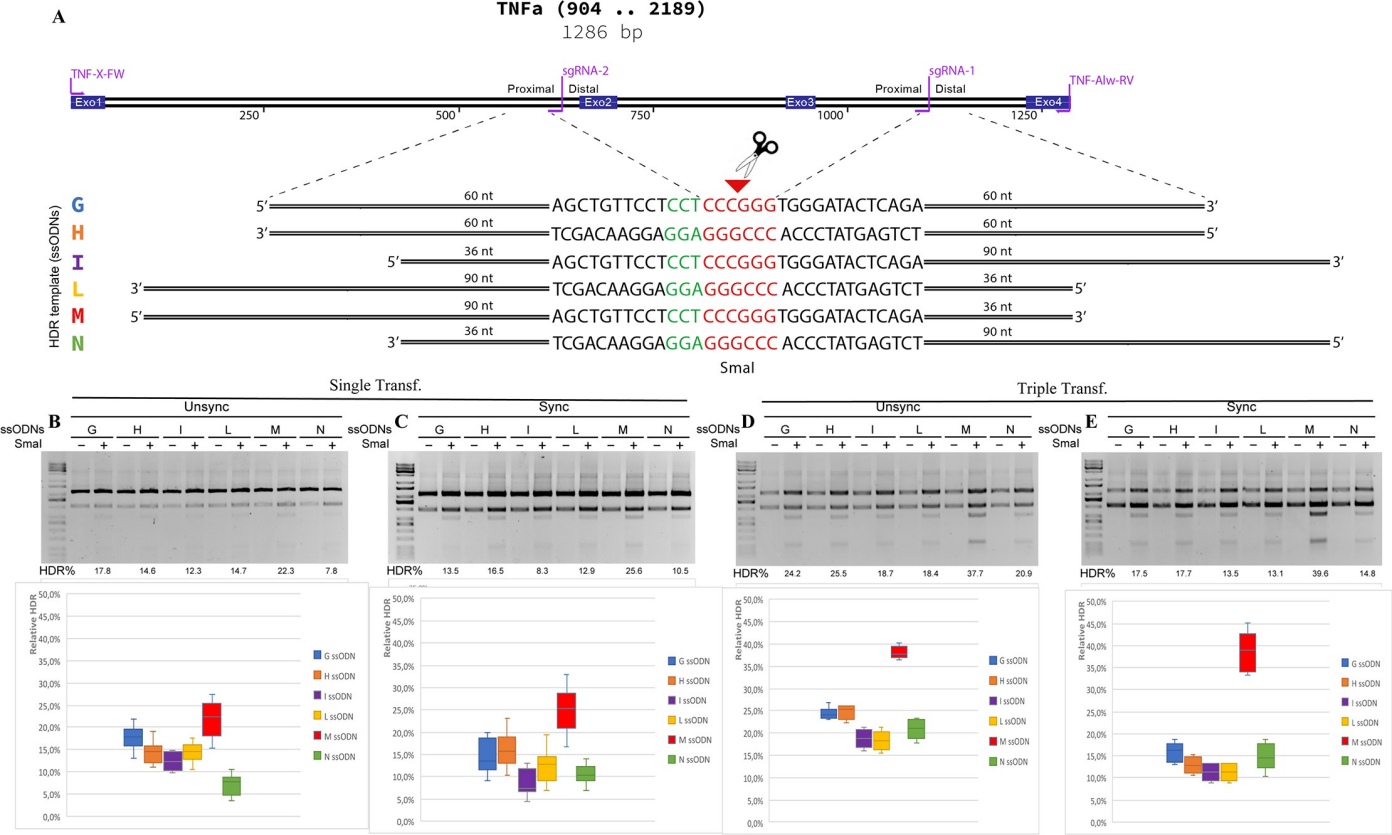
Systematic investigation of DNA templates, and two sgRNA, for efficient HDR at the *TNFα* locus in HEK293 cells. (**A**) A segment of human *TNFα* shows the genome structure, the two sgRNA 2 guides (sgRNA1, sgRNA2) sites, and the primer used for PCR amplification (in violet). +1: ATG Six HDR templates (color-coded) were tested for HDR efficiency, the PAM region (in green). Template ssODNs contains SmaI restriction sites (in red) that are flanked by various lengths of homology arms. (**B**) HDR efficiency was tested in single and triple transfection, in combination with synchronized cells. The mean % HDR and standard deviation (error bar) were determined by SmaI digestion from eight and nine replicates. Representative gels from PCR and HDR analyses are shown for each cell condition.

**Fig 6:**
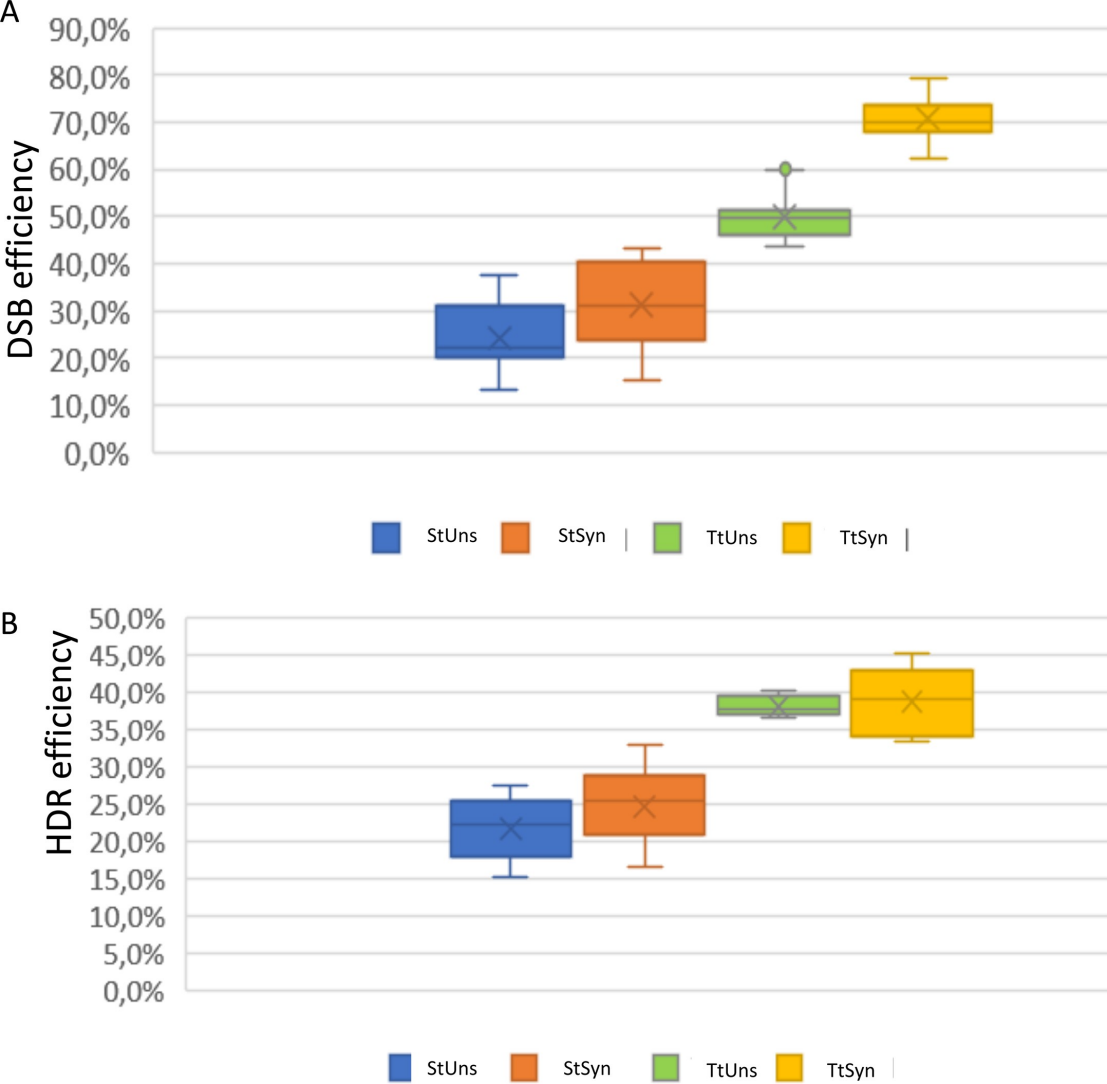
DSB efficiency (A) Editing efficiency tested on four different conditions. The DSB efficiency increases with triple transfection in synchronized cells (TtSyn). **(B)** Double cut strategy and donor M, in single and triple transfection, in synchronized and unsynchronized cells. The triple transfection increased HDR efficiency. StUns = Single transfection method, unsynchronized cells; StSyn = Single transfection in synchronized cells; TtUns = Triple transfection method in unsynchronized cells; TtSyn = Triple transfection in synchronized cells.

Furthermore, we tested the HDR efficiency combining the use of dual 2sgRNA and rational design of ssODNs. HEK293 cells have been co-transfected with the pX333-2gRNA1/2 guide (which induces double DSB) and six ssODNs (G-N) (Fig 5A). Subsequently, we systematically determined the effect on HDR efficiency in controls, in nocodazole synchronized cells, and triple transfection. To note, as already here described, a simple PCR amplification before the SmaI digestion (that shows the DNA deletions caused by 2sgRNA1/2 was sufficient to assess the DSB efficiency under various experimental conditions. Interestingly, the DSB efficiency increased more than three-fold with triple transfection in synchronized cells (Fig 6A).

### Dual cut and HDR efficiency

We further compared the HDR efficiency using pX333sgRNA1/2 and the six ssODNs (G-N) with SmaI digestion (Fig 5B to 5E). With 2sgRNA1/2, all ssODNs designed showed detectable SmaI digestion bands that indicated the HDR occurrence in all experimental conditions used. The HDR efficiency increased with triple transfection for all ssODNs. The donor ssODN M had the same design as donor E (the most efficient donor in the single cut comparison), with asymmetric arms complementary to a non-target locus with 90bp on the PAM-proximal side and 36bp extension arm on the PAM-distal side of the break [12], achieving an HDR efficiency up to 39% (Fig 5B to 5E). The graphic in S2 Fig shows the effect of nocodazole and triple transfection on HDR efficiency using double sgRNA guides. Again, the nocodazole did not significantly affect the HDR; the triple transfection increased the HDR for all donors by nearly 1,3–2,6 fold. The nocodazole diminished the HDR efficiency except for donor M. The graphic in Fig 6B compares the HDR efficiency of donor M in all experimental conditions (Fig 6B). The HDR, after triple transfection in sync cells, increased up to 1,8-fold. As already reported in the literature, even in the *TNFα* locus, the use of 2sgRNA dramatically increased the HDR compared to single sgRNA more than double [1].

Our results demonstrated, for the first time, that the combination of 2sgRNA, asymmetric donor, and triple transfection, induced a dramatic rise of HDR, from undetectable HDR event to 39% HDR efficiency.

### Off-targets analyses

To predict the most likely off-target sites for the sgRNAs employed to edit the *TNFα* gene in this study, we used a public webserver: (https://eu.idtdna.com/site/order/designtool/index/CRISPR_PREDESIGN) able to assess and prioritize potential CRISPR/Cas9 activity at off-target loci. The T7E1 assay evaluated the top three potential off-target sites for each sgRNA. None of the loci analyzed showed detectable levels of off-target events (S3B Fig).

## Discussion

Here, we reported a new and straightforward approach to enhancer genome engineering in human cells. We proved, for the first time, that the simultaneous use of double sgRNA, asymmetric donor, and triple transfection increased the HDR efficiency.

First, we compared the performance of single sgRNA with coupled 2sgRNA to edit the *TNFα* locus. According to data already reported in literature, our results revealed that the use of 2sgRNA increases the DSB efficiency [1] dramatically. Furthermore, the management of 2sgRNA created a precise DNA excision between each PAM sequence, a massive advantage over the randomly sized indels generated by single sgRNA transfection (Fig 2). The deletions between the 2sgRNA could be easily identified by PCR amplification and agarose electrophoresis, thus avoiding the T7E1 assay. Besides, the results confirmed that the DSB grew after triple transfection up to two-fold and, combined with nocodazole treatment, up to three-fold (Fig 6).

As already known, even in the *TNF*α locus, the use of 2sgRNA raised the HDR efficiency more than double compared to transfection of single sgRNA. This data could be explained by the capability of double 2sgRNA to create a precise cut on the target site, oscillating from time to time only for a few bases, without generating unpredictable indels. Otherwise, a single-cut approach would induce truly extensive deletions reducing significantly the possibility of binding the homology arms, leading to a drastic drop in HDR rates.

We then tested the HDR frequency by transfecting various ssODN molecules with different structures combined with single sgRNA and 2sgRNA pair. In particular, with the method of single sgRNA, we observed that donor DNA complementary to the non-target strand were more effective than the ones complementary to the target strand, and this was consistent with previous studies performed with various ssODNs structure in human cells [12], or using symmetric ssODNs to introduce mutations at the EMX1 and AAVS1 loci in human cell lines [5, 23] (Fig 3). With the 2sgRNA pair, since all ssODNs had a higher HDR efficiency, this effect was less marked but still present (Fig 5).

Our results also showed, again in line with Richardson at al., that the asymmetric ssODN donor complementary to the non-target strand with the arm of 36bp on the PAM-distal side and the 90bp arm on the PAM-proximal site of the break had the highest HDR efficiency using single sgRNA, and the couple 2sgRNA (donor E, M) [12]. The asymmetric donor allowed the shorter arm to bind to the distal PAM early-released strand and the longer to bind to the PAM proximal portion of the non-target strand by strand intrusion and complementary strand displacement. This donor structure increased the HDR by about two-fold.

In contrast with literature data, we noted that the treatment with nocodazole did not develop HDR efficiency [5]. Lin et al. presented a systematic study of nocodazole concentration on HDR efficiency. They transfected the cells after 17h of the nocodazole treatment at a 200 ng/ml concentration. They determined that nocodazole increased the HDR efficiency when used with a low concentration of Cas9 (30pmol), while 100 pmol diminished the enhancement. It is plausible to think that since we transfected a plasmid which induced Cas9 expression to high dosage in our experimental conditions, we did not apply the correct Cas9 concentration to induce an improvement in HDR efficiency. Moreover, we used 100 ng/ml of nocodazole that was added four hours after the transfection. In our experiments, the nocodazole effect was detected only on the donor E and M, the best donor structures, also capable of increasing HDR efficiency in cells synchronized with a wrong Cas9 concentration.

We showed that the triple transfection increased the HDR from 1,3–2,6 fold. Moreover, our results attested, for the first time, that the highest HDR efficiency was achieved when 2sgRNA, the asymmetric donor (M), and triple transfection in synchronized cells were utilized in the same experiment (Fig 3E). With this system, we maximized HDR’s efficiency, from undetectable HDR events (single guide, symmetric donor, unsynchronized cells, with single transfection) to 39% HDR efficiency. We also proved that the *TNFα* gene could be edited with CRISPR/Cas9 methodology with extraordinary efficiency. These data concern a single locus and demonstrates a higher efficiency of homologous recombination for TNF*α* locus, further investigations, on multiple loci, are still needed to fully characterize the data here reported.

Finally, our work outcomes could be used as a guideline to improve the efficiency (and the utility) of CRISPR/Cas9-mediated genome engineering through the most effective optimizations to date.

## Supporting information

S1 Fig Single cut strategy with sgRNA2 guide. **(A,B)** HDR efficiency between synchronized and non-synchronyzed cells, in single and triple transfection, respectively. **C,D** HDR efficiency between single and triple transfection in synchronyzed and unsynchronized cells. The figure contains a single dot for each experimental value obtained (short lines), and median value is reported (long lines).
(TIF)

S2 Fig Double cut strategy with 2sgRNA1/2 guide. **(A,B)** HDR efficiency between synchronized and non-synchronyzed cells in single and triple transfection, respectively. **C,D** HDR efficiency between single and triple transfection in synchronyzed and unsynchhronized cells. The figure contains a single dot for each experimental value obtained (short lines), and median value is reported (long lines).
(TIF)

S3 Fig **(A)** Sanger Sequence that showed the correct HDR events. **(B)** Evaluation of CRISPR/Cas9 off-target effects for sgRNAs designed to knock-out the human *TNFα* gene. T7 assay analysis at the top three potential off-target sites and the first potential genic off-target site in HEK293T cells. OT: off-target locus.
(TIF)

S1 Materials (ZIP)

S1 Raw data (XLSX)
