# Supplementary figures and images for: Systematic analysis of factors that improve homologous direct repair (HDR) efficiency in CRISPR/Cas9 technique
Source: PLoS One. 2021 Mar 5;16(3):e0247603. doi: 10.1371/journal.pone.0247603 (PMC7935300; doi:10.1371/journal.pone.0247603)

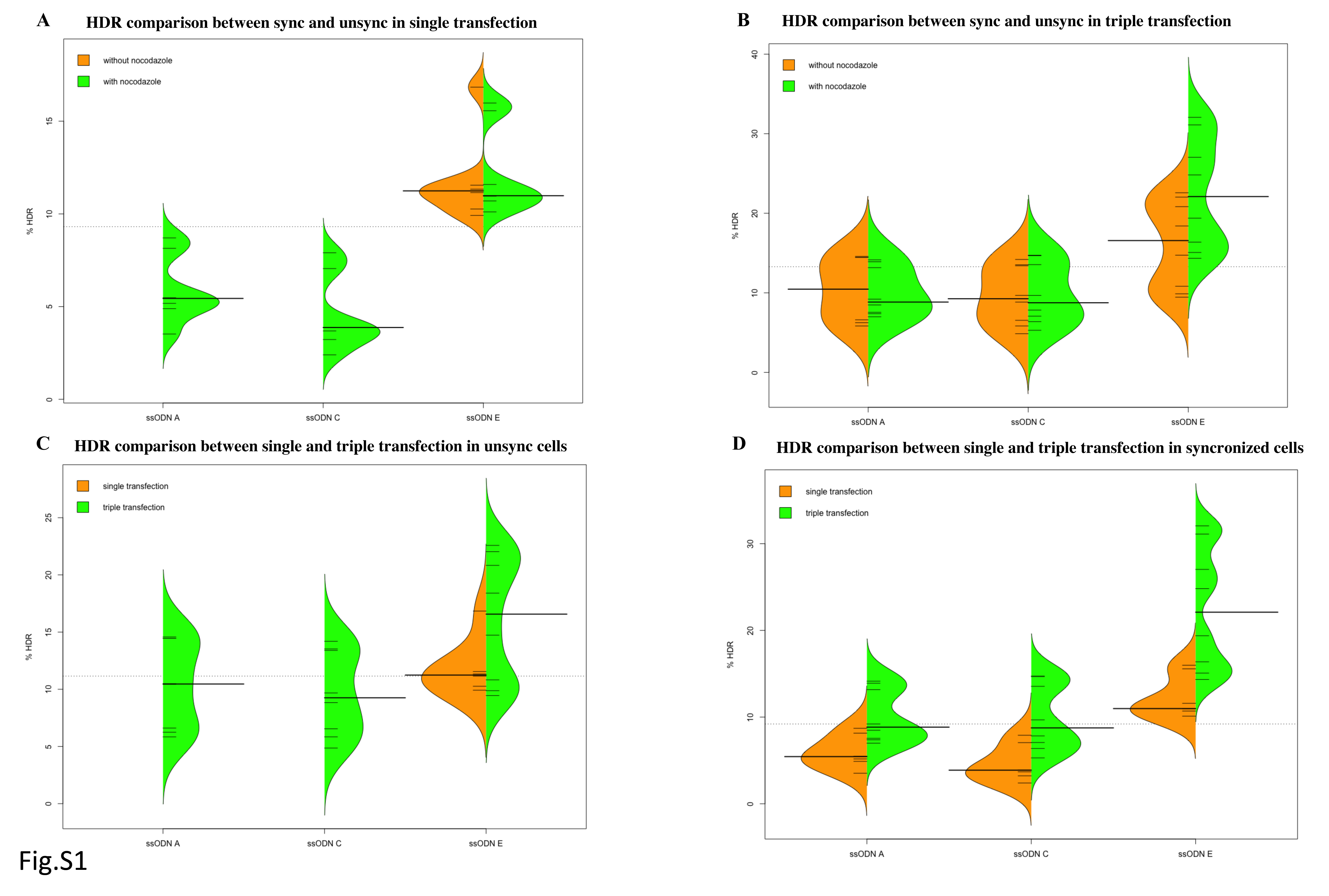

Supplement: S1 Fig — (A,B) HDR efficiency between synchronized and non-synchronyzed cells, in single and triple transfection, respectively. C,D HDR efficiency between single and triple transfection in synchronyzed and unsynchronized cells. The figure contains a single dot for each experimental value obtained (short lines), and median value is reported (long lines). (TIF) [file pone.0247603.s001.tif]

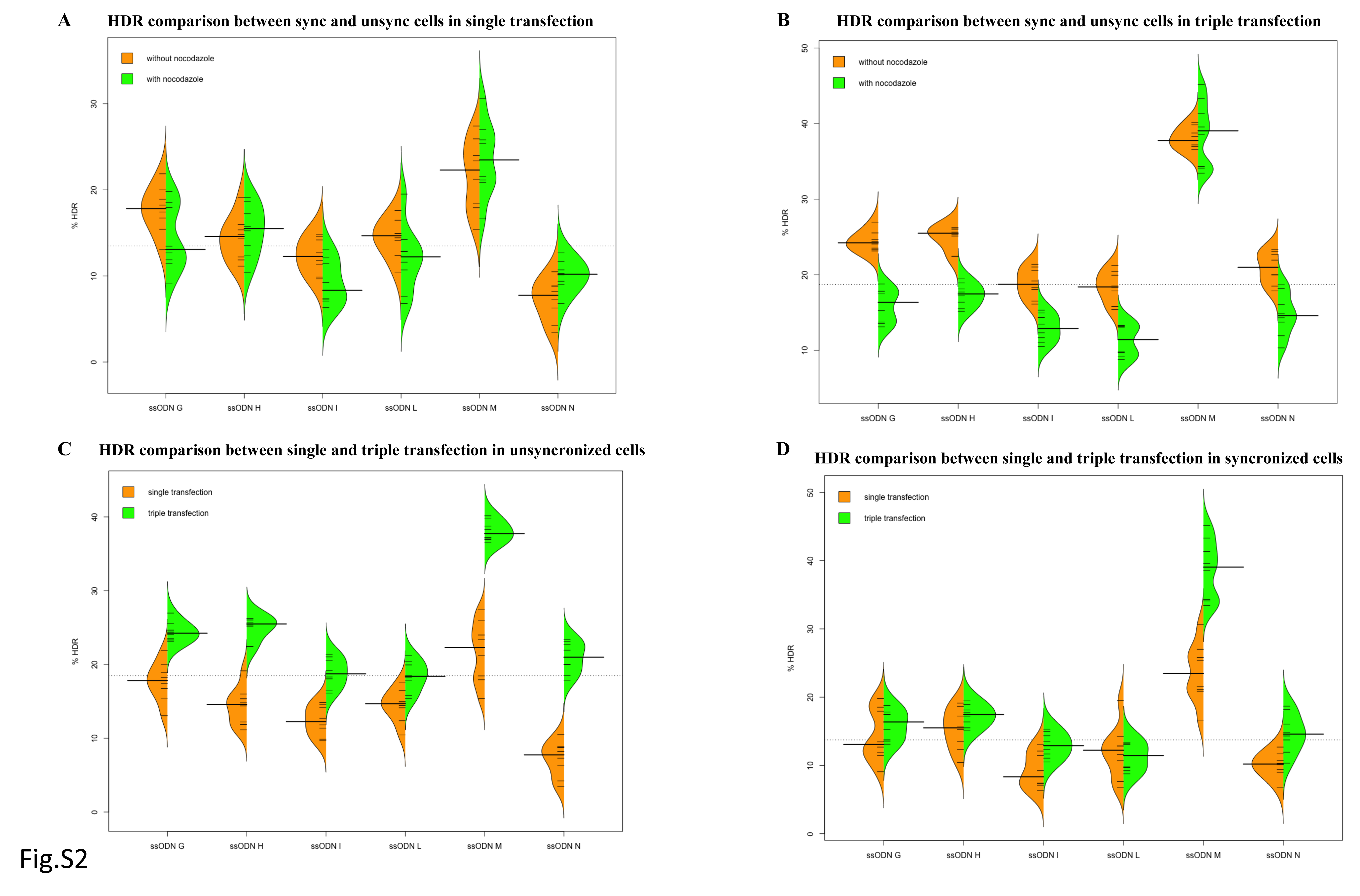

Supplement: S2 Fig — (A,B) HDR efficiency between synchronized and non-synchronyzed cells in single and triple transfection, respectively. C,D HDR efficiency between single and triple transfection in synchronyzed and unsynchhronized cells. The figure contains a single dot for each experimental value obtained (short lines), and median value is reported (long lines). (TIF) [file pone.0247603.s002.tif]

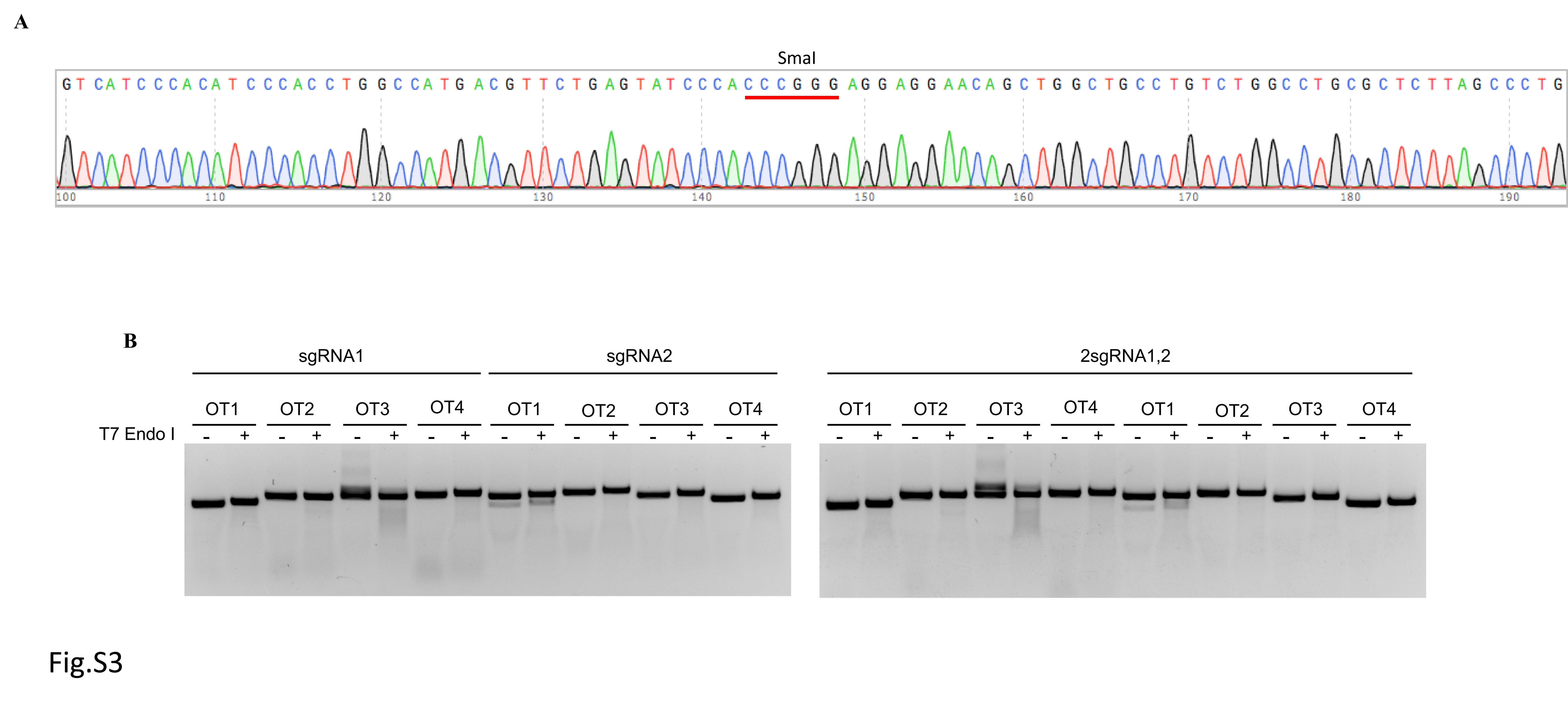

Supplement: S3 Fig — (A) Sanger Sequence that showed the correct HDR events. (B) Evaluation of CRISPR/Cas9 off-target effects for sgRNAs designed to knock-out the human TNFα gene. T7 assay analysis at the top three potential off-target sites and the first potential genic off-target site in HEK293T cells. OT: off-target locus. (TIF) [file pone.0247603.s003.tif]

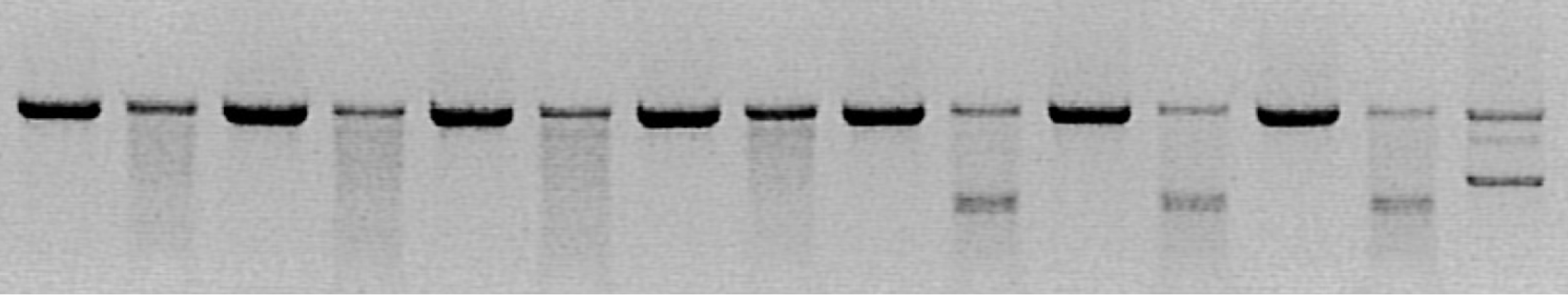

Supplement: S1 Materials — (ZIP) [file pone.0247603.s004.zip › untitled folder/2A.tif]

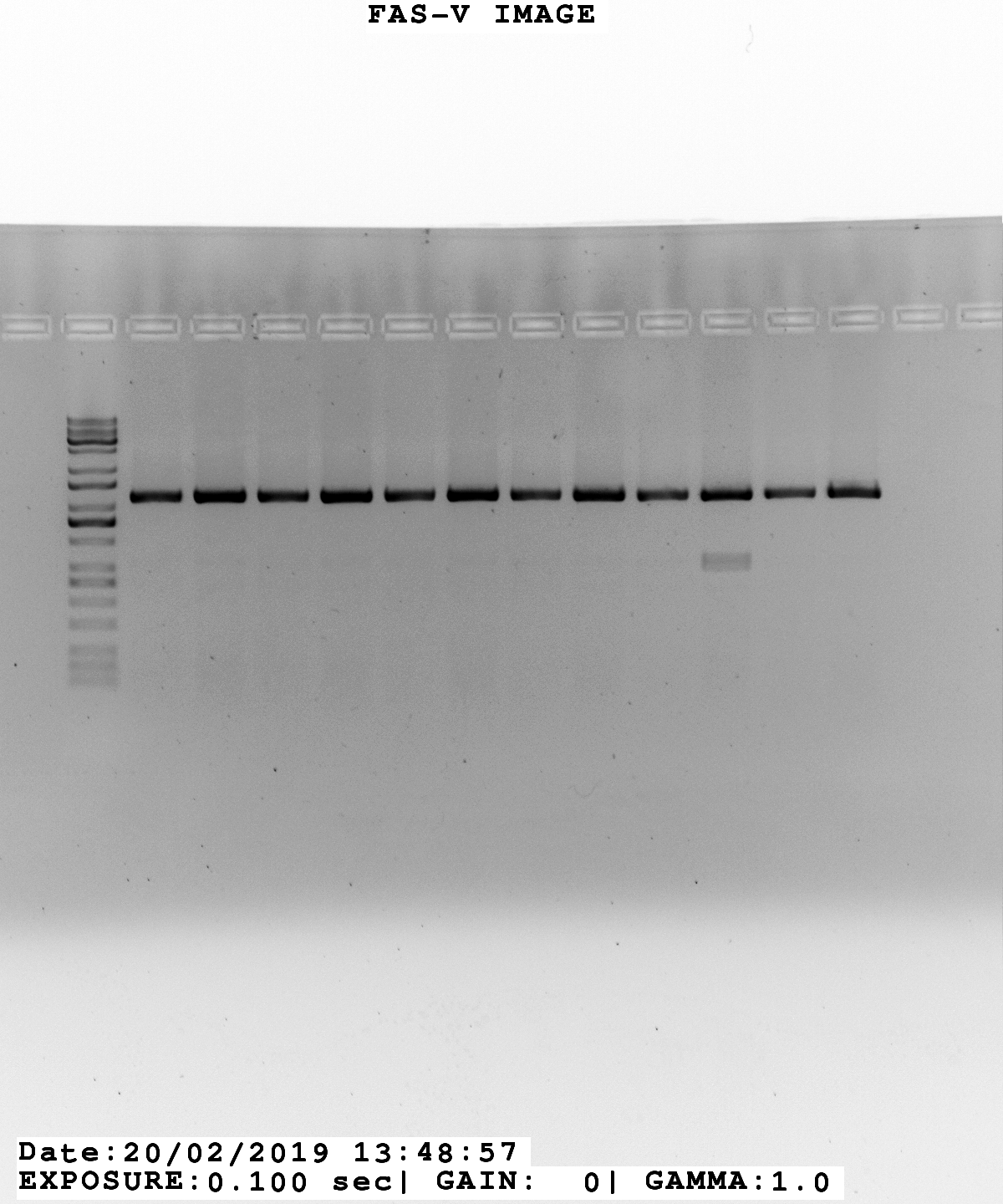

Supplement: S1 Materials — (ZIP) [file pone.0247603.s004.zip › untitled folder/3B.tif]

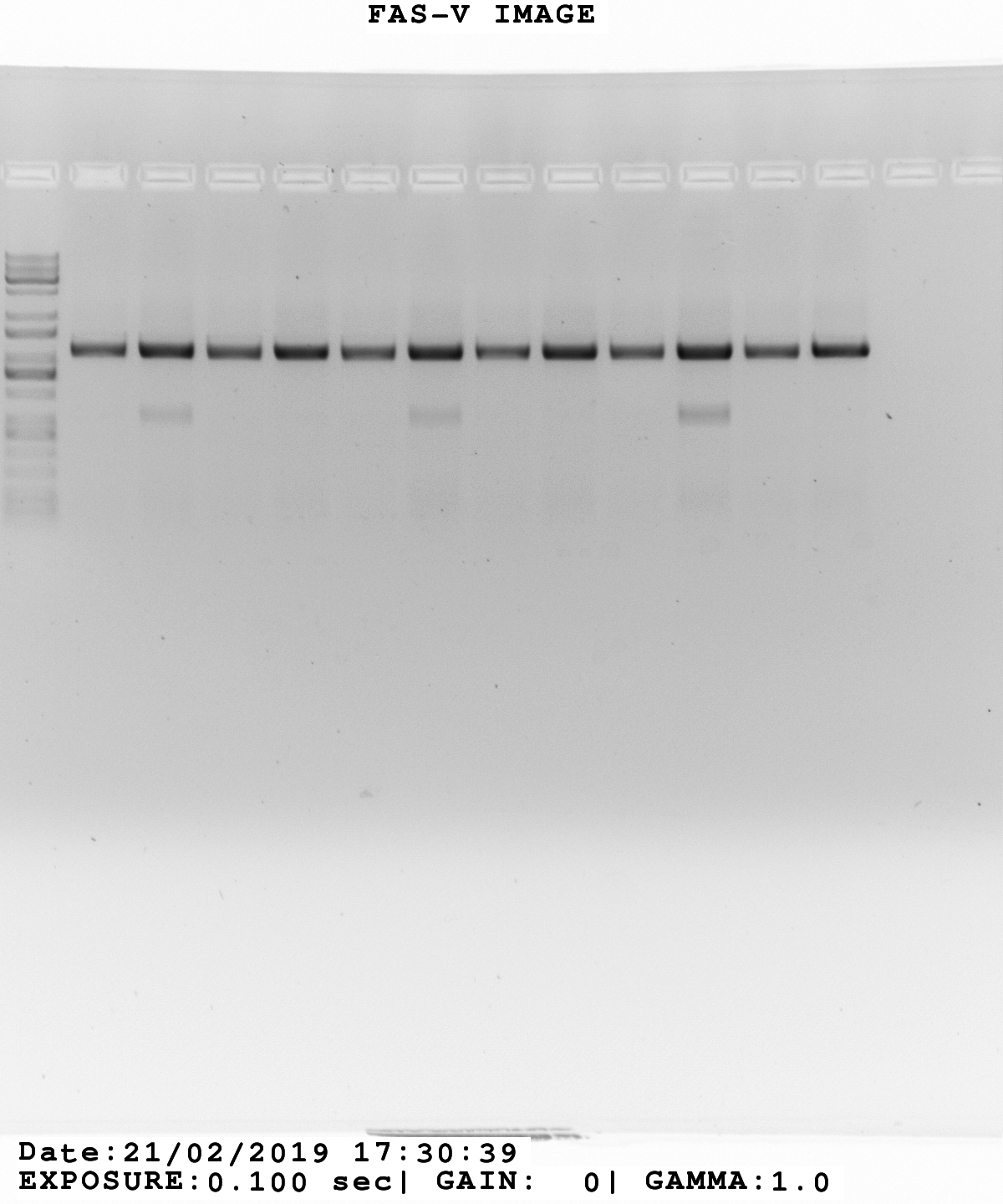

Supplement: S1 Materials — (ZIP) [file pone.0247603.s004.zip › untitled folder/3C.tif]

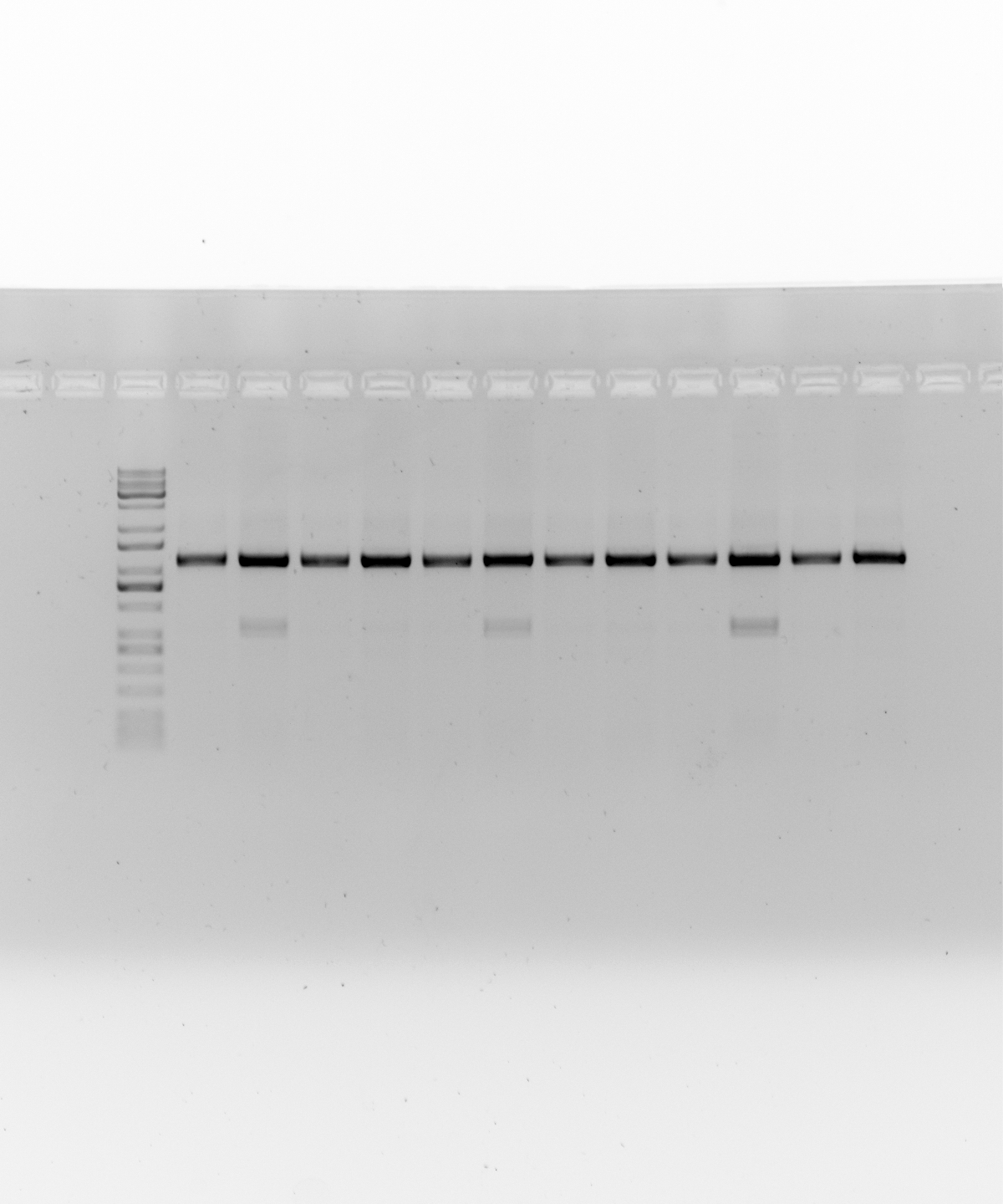

Supplement: S1 Materials — (ZIP) [file pone.0247603.s004.zip › untitled folder/3D.tif]

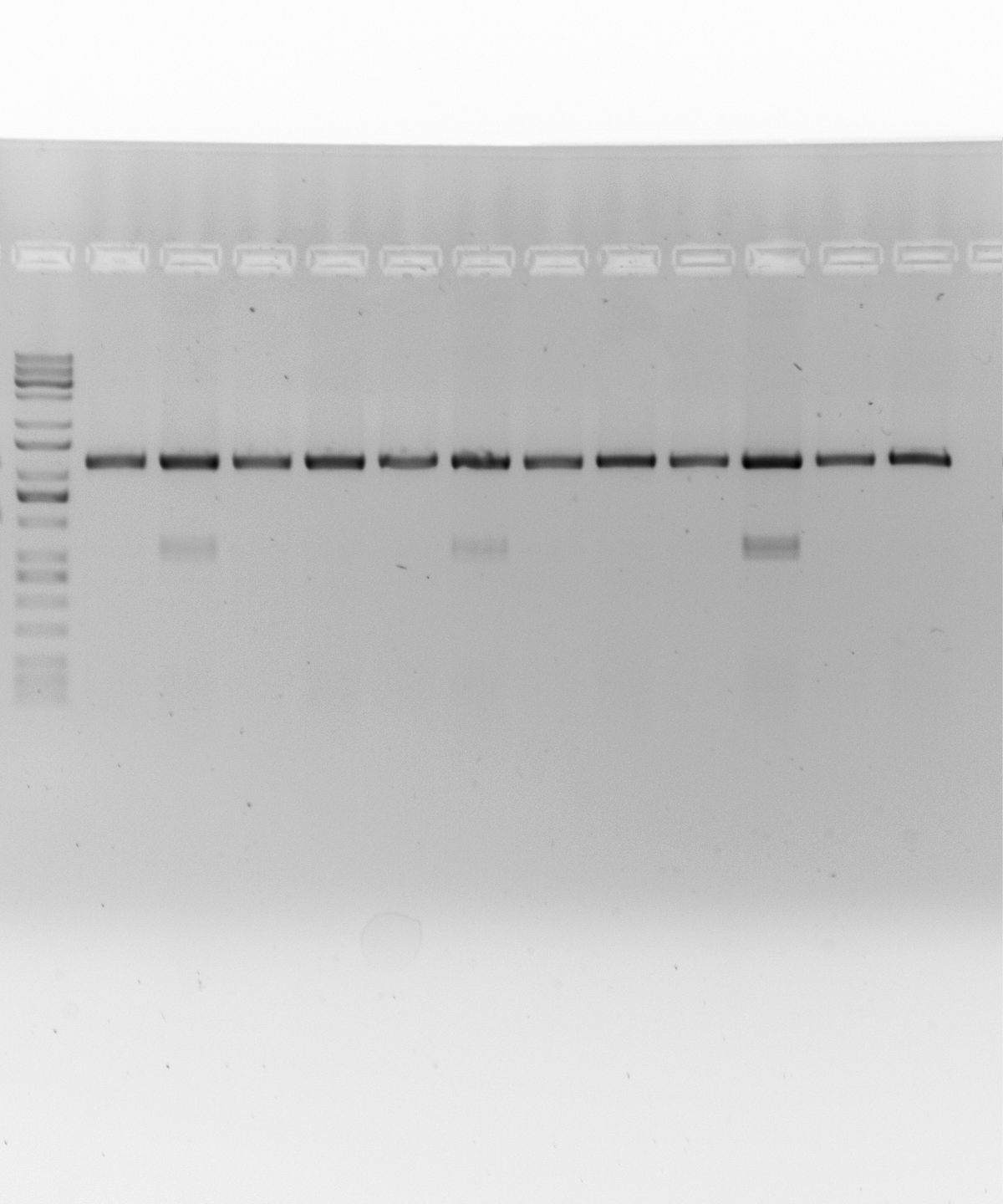

Supplement: S1 Materials — (ZIP) [file pone.0247603.s004.zip › untitled folder/3E.tif]

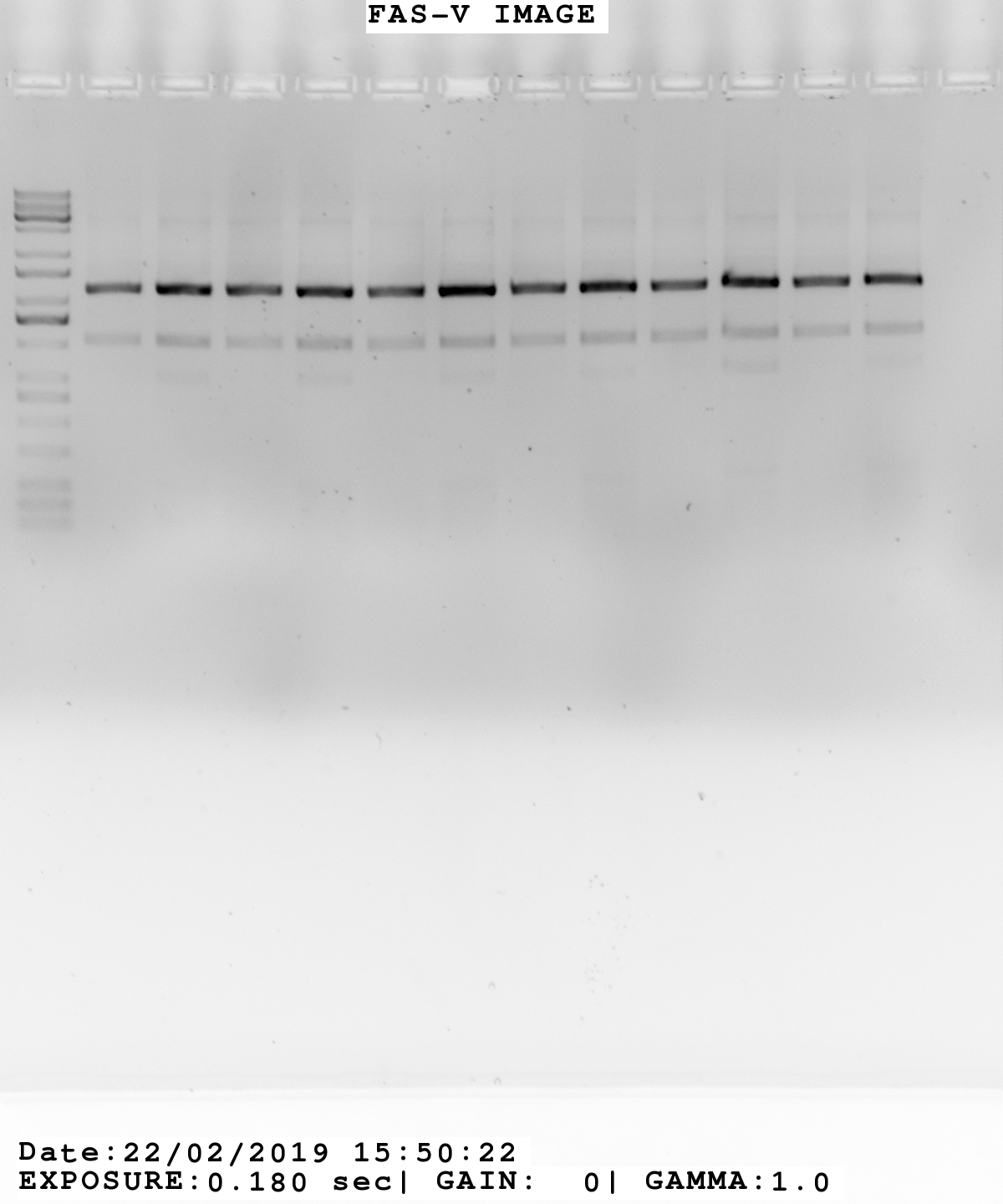

Supplement: S1 Materials — (ZIP) [file pone.0247603.s004.zip › untitled folder/5B.tif]

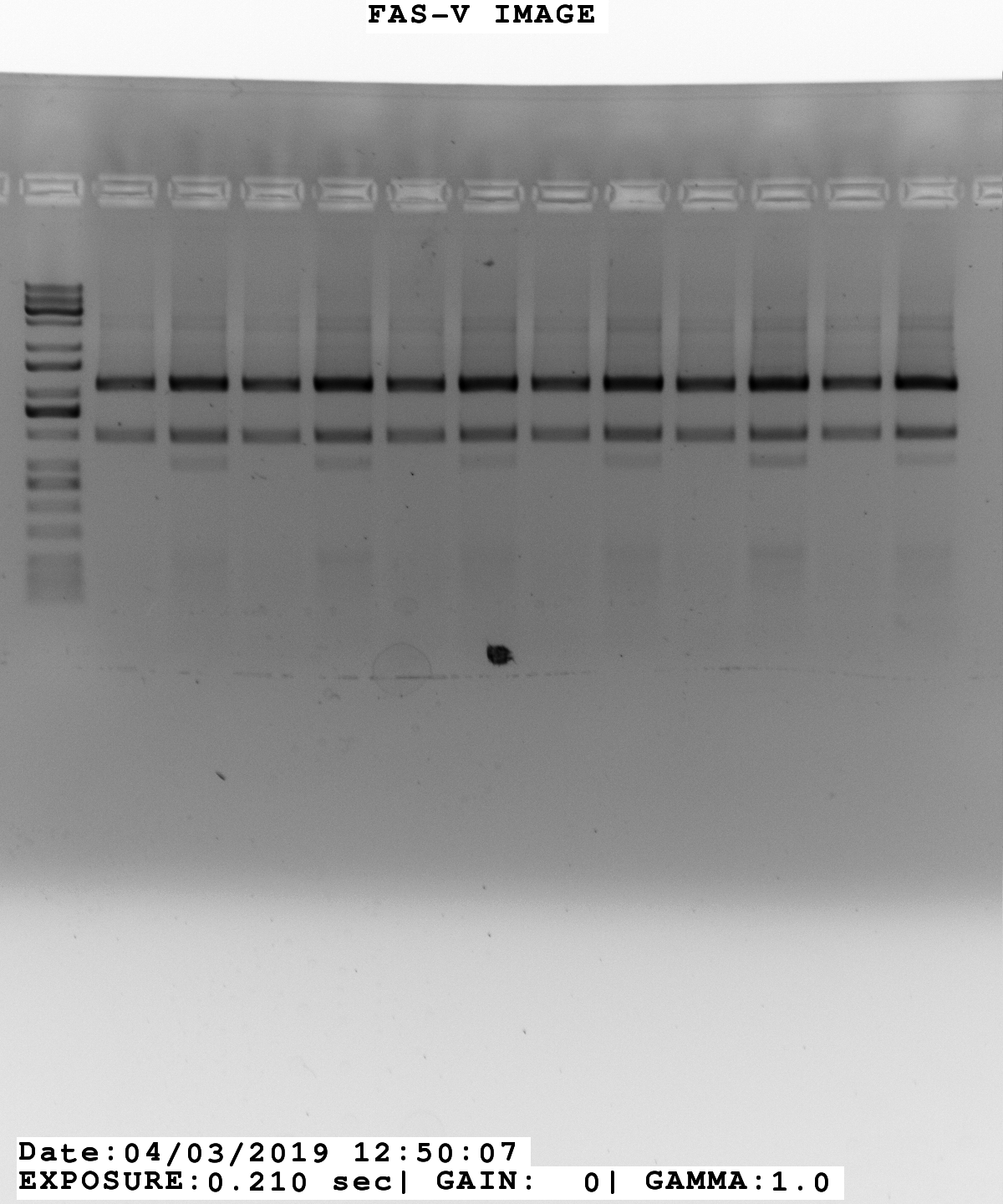

Supplement: S1 Materials — (ZIP) [file pone.0247603.s004.zip › untitled folder/5C.tif]

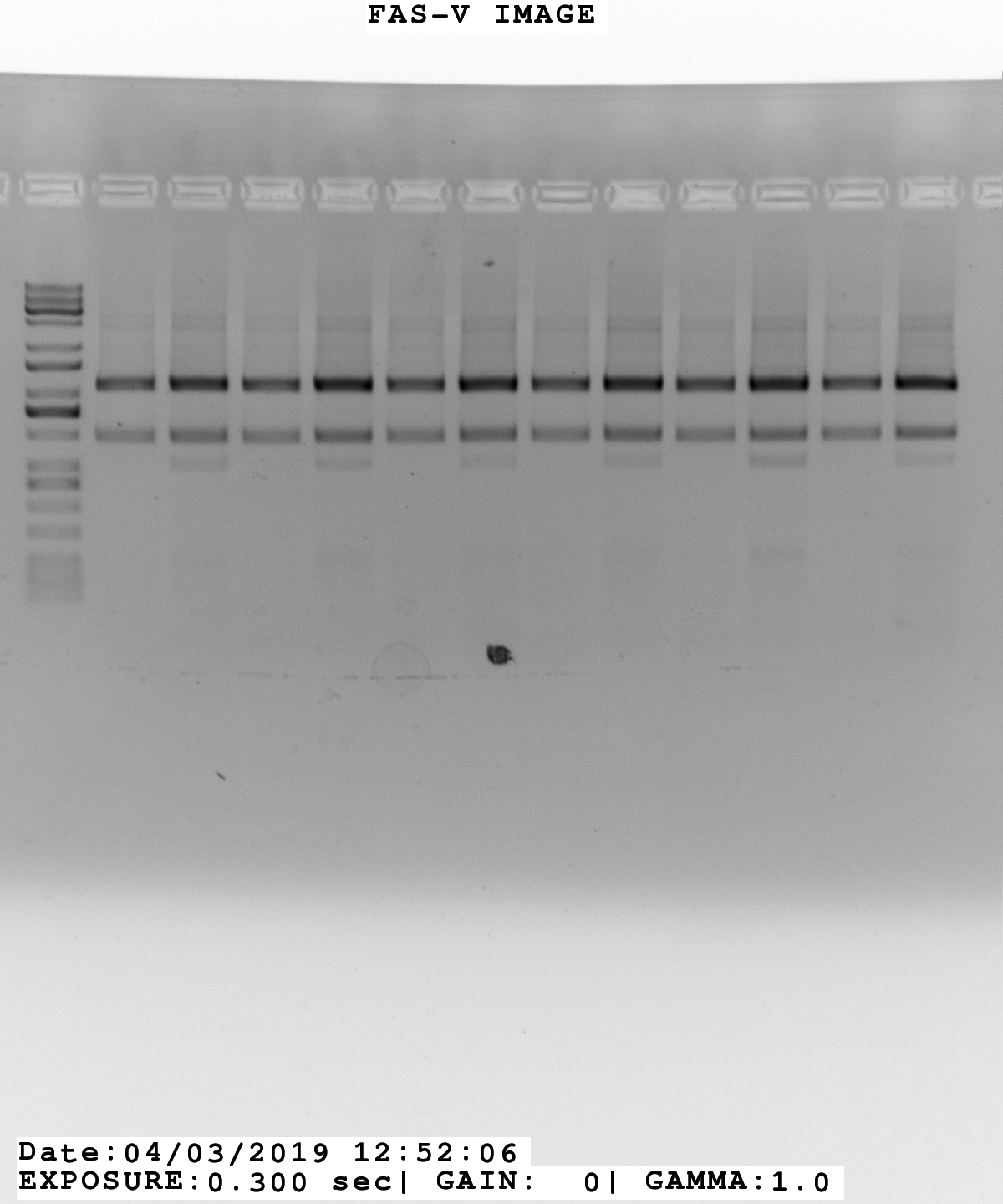

Supplement: S1 Materials — (ZIP) [file pone.0247603.s004.zip › untitled folder/5D.tif]

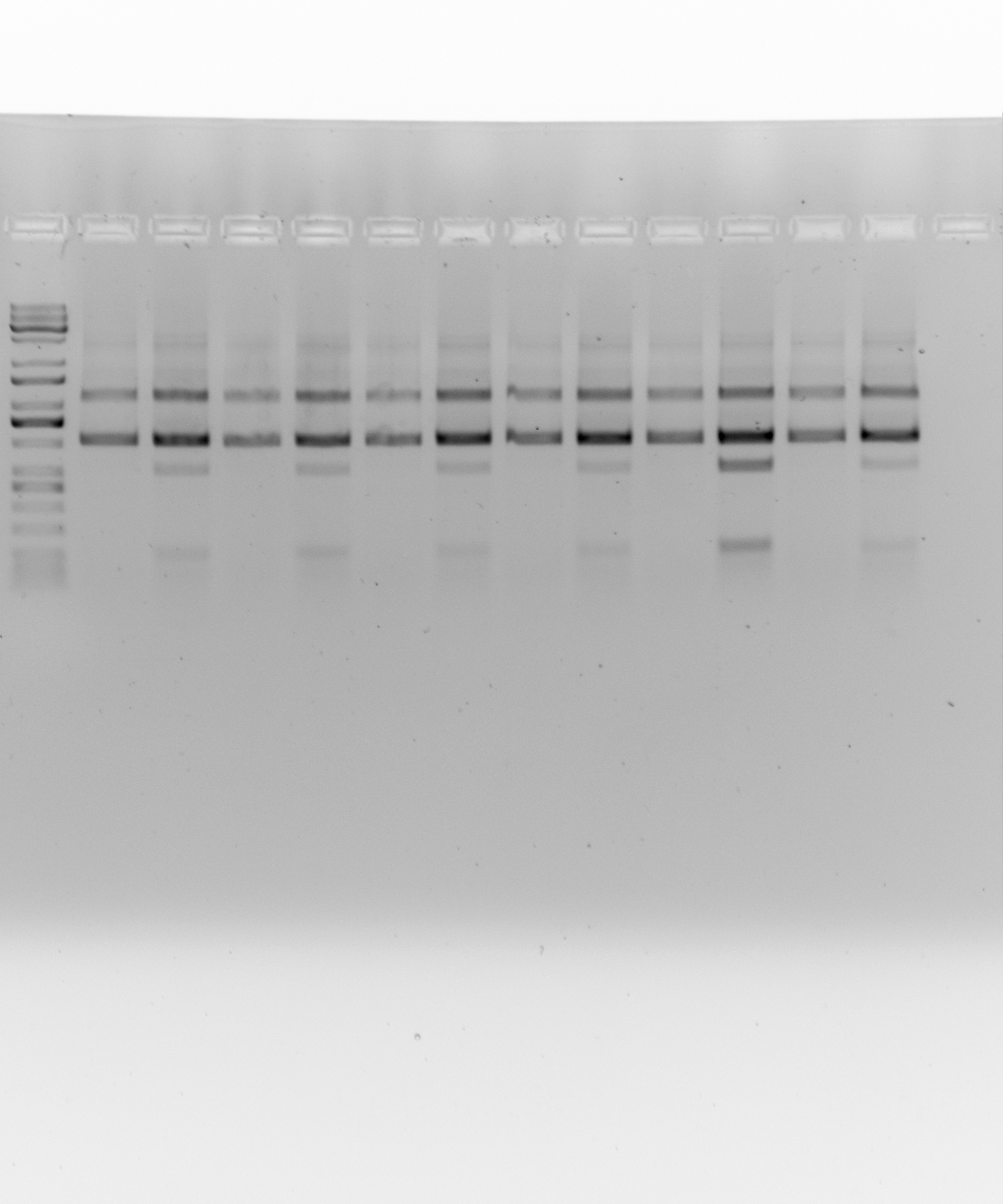

Supplement: S1 Materials — (ZIP) [file pone.0247603.s004.zip › untitled folder/5E.tif]

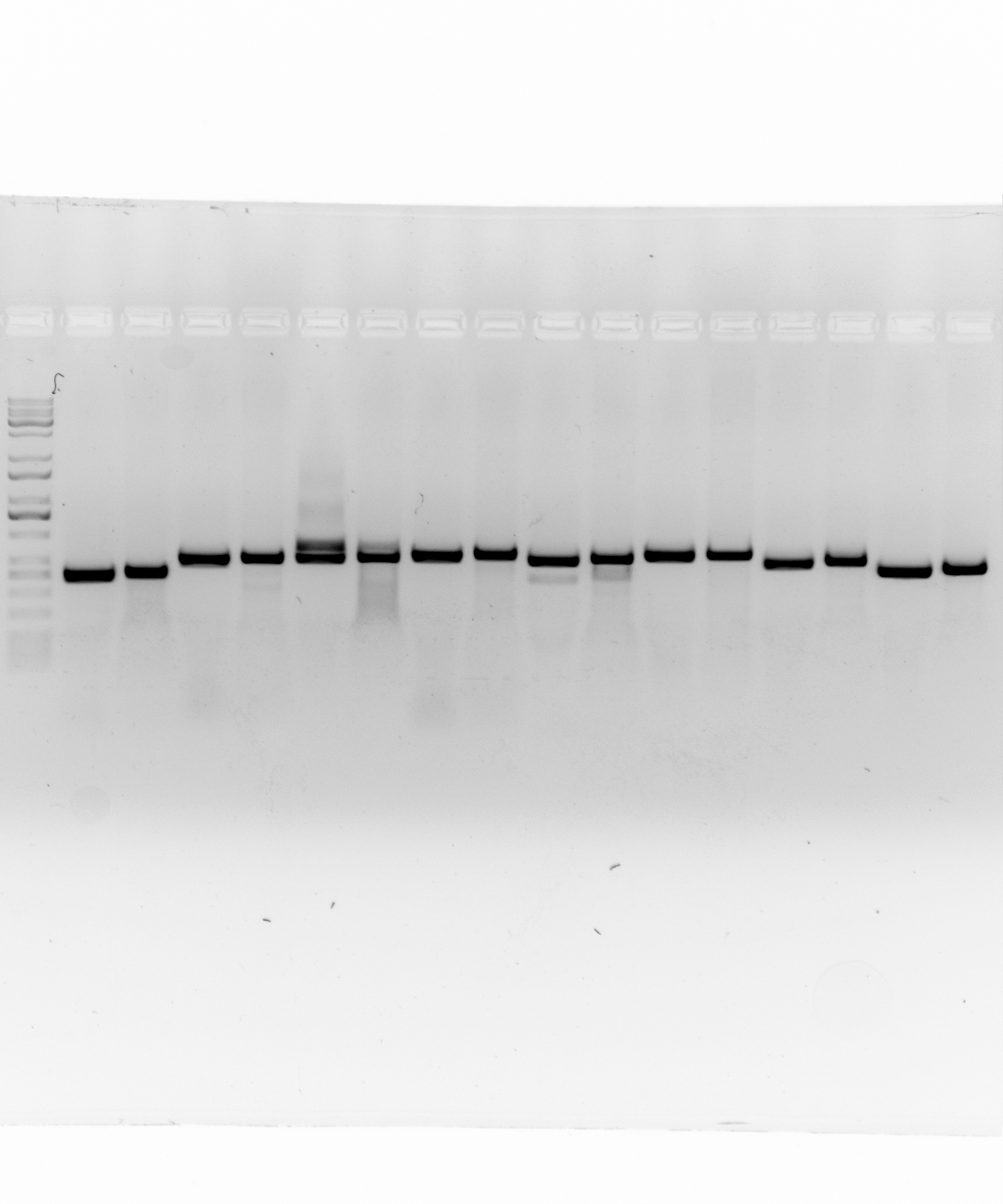

Supplement: S1 Materials — (ZIP) [file pone.0247603.s004.zip › untitled folder/S3 B.tiff]

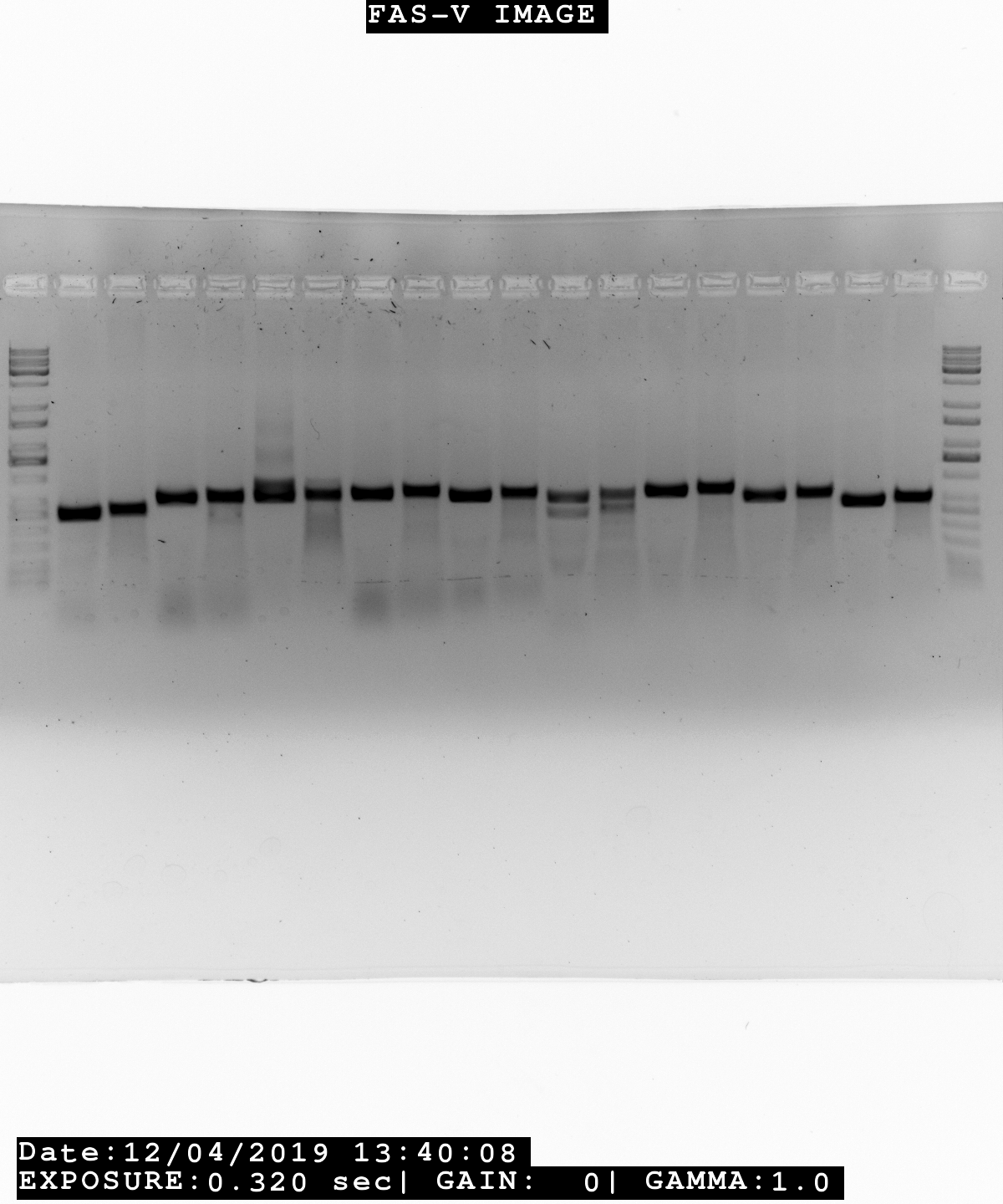

Supplement: S1 Materials — (ZIP) [file pone.0247603.s004.zip › untitled folder/S3.tif]
